# Ajuba receptor mediates the internalization of tumor-secreted GRP78 into macrophages through different endocytosis pathways

**DOI:** 10.18632/oncotarget.24090

**Published:** 2018-01-09

**Authors:** Xiaoqin La, Lichao Zhang, Hanqing Li, Zhuoyu Li, Guisheng Song, Peng Yang, Yufei Yang

**Affiliations:** ^1^ Institute of Biotechnology, Key Laboratory of Chemical Biology and Molecular Engineering of National Ministry of Education, Shanxi University, Taiyuan 030006, China; ^2^ Institute of Biomedical Sciences, Shanxi University, Taiyuan 030006, China; ^3^ School of Life Science, Shanxi University, Taiyuan 030006, China; ^4^ Department of Bioengineering and Therapeutic Sciences, University of California, San Francisco, CA 94143, USA

**Keywords:** tumor-secreted GRP78, macrophages, endocytosis, Ajuba, tumor microenvironment

## Abstract

Glucose-regulated protein 78 (GRP78), an ER chaperone, is overexpressed in cancer cells. Solid tumor cells can secrete GRP78 that can promote tumor angiogenesis, differentiation of bone marrow-derived mesenchymal stem cells, tumor cell proliferation and polarization of tumor-associated macrophages. However, the mechanism by which GRP78 functions as a tumor promoter either by staying on the membrane to stimulate intracellular signals or directly entering into cytosolic remains unknown. Here, we reported that an endotoxin-free His-GRP78 protein was purified *in vitro* that simulates original secreted GRP78. Through analyzing GRP78 concentration in serum samples from 32 colon cancer patients, 40 nM His-GRP78 was selected as an optimized dose to treat cells. Biochemical analysis revealed that secreted GRP78 was able to enter into RAW264.7 and THP-1 cells directly rather than stay on the plasma membrane to transfer signals. Further studies showed that GRP78 internalization was endocytosis-dependent, and both phagocytosis and clathrin, caveolin-1 and micropinocytosis-mediated endocytosis pathways contributed to internalization of secreted GRP78 into cells. Mechanistically, Ajuba is able to interact with GRP78. Ablation of Ajuba suppressed the internalization of secreted GRP78 into cells, indicating that Ajuba was responsible for internalization of secreted GRP78 into RAW264.7. Furthermore, we observed that internalized GRP78 could entered into the mitochondrion and endoplasmic reticulum, which provided a suitable place and enough time for GRP78 to function in molecular and cellular processes. Together, these results reveal a novel mechanism by which secreted GRP78 internalizes into macrophages in the tumor microenvironment, which provides a potential target for drug development.

## INTRODUCTION

Glucose-regulated protein 78 (GRP78), an immunoglobulin heavy chain binding protein (Bip), is a member of the HSP70 protein family. It is well established that GRP78 is an endoplasmic reticulum (ER) chaperone and regulator of ER stress signaling [1, 2]. It is widely overexpressed in solid tumors induced by microenvironmental factors such as hypoxia, acidosis and glucose deficiency [3]. High levels of GRP78 contribute to the acquisition of cancer hallmarks, including apoptosis resistance, immune escape, metastasis and angiogenesis [4]. In addition to localizing in the ER, GRP78 is present in the cytoplasm, mitochondria , plasma membrane and nucleus of tumor cells [5]. Particularly, GRP78 is also secreted by tumor cells, and plays important roles in tumorigenesis [6–10]. Tumor-secreted GRP78 can block the antiangiogenic activity of bortezomib through inducing pro-survival signals via phosphorylation of extracellular signal–related kinase and inhibiting p53-mediated expression of pro-apoptotic Bok and Noxa proteins in endothelial cells, and it can differentiate bone marrow-derived mesenchymal stem cells (BMSCs) into cancer-associated fibroblasts (CAFs) through activating TGF-β/Smad signaling pathway in an autocrine/paracrine manner [8, 10]. All these findings imply that secreted GRP78 has a powerful transform function for tumor microenvironment (TME). Moreover, considering that the hallmarks of cancer have also emphasized the importance of the tumor microenvironment during tumorigenesis and invasion [11], the study on how secreted GRP78 influences TME will play important roles in elucidating mechanism of tumorigenesis.

It is known that the immune system is a major player to modulate tumor microenvironment [12]. Tumor-associated macrophages (TAMs) are activated in tumors and function as a promotor of tumor progression [13]. Our previous study showed that secreted GRP78 polarized TAMs to M2 type. However, the function of tumor-secreted GRP78 in macrophages remains unclear. Therefore, we will focus on (1) secreted GRP78 functions effectively by staying on the cell surface to provoke intracellular signals or directly entering into cells to activate intracellular signals; and (2) which endocytic pathway(s) controls internalization of GRP78 if it can get into cells; and (3) which organelle(s) entered GRP78 locates in.

Endocytosis is the predominant manner of macromolecules and particles uptake in eukaryotic cells [14]. During endocytosis, the plasma membrane invagination and forms vesicles, also called endocytic vesicles. According to the different molecular mechanisms and the sizes of endocytic vesicles formation, the endocytosis is divided into phagocytosis and pinocytosis. The diameter of phagocytic vesicles is often more than 250 nm, and the diameter of pinocytic vesicles is generally less than 150 nm. Phagocytosis mainly occurs in macrophages. In this process, the foreign substances interact with cell-surface receptors and are wrapped into cell by protruded pseudopodia [15]. Different from phagocytosis, pinocytosis is generally divided into three categories: clathrin-mediated endocytosis, caveolin-mediated endocytosis and micropinocytosis, which occurs in almost all of the eukaryotic cells [16]. Clathrin-mediated endocytosis form clathrin and adaptor proteins-coated vesicle to translocate xenobiotics from extracellular to intracellular, and this process is cell-surface receptor-dependent [17]. Caveolae-mediated endocytosis needs the formation of flask-shaped invagination in lipid raft regions of the plasma membrane with caveolin proteins (caveolin-1, -2, -3) presence, then the invagination pack macromolecules to form endosomes to achieve internalization of the particles [18]. Macropinocytosis involves a non-selective uptake of extracellular fluid and particles by actin-dependent invaginations of the plasma membrane and leads to the formation of large, uncoated endocytic vesicles typically range from 0.2 to 10 μm [19, 20]. Of note, in all of the different types of endocytosis mentioned above, the endosomes are regarded as the sorting stations, which mediating macromolecules entering the cells into different organelles such as mitochondria, endoplasmic reticulum, Golgi, Lysosomes, nuclei, etc.

In this study, we observed that tumor-secreted GRP78 could enter into RAW264.7 and THP-1 cells and the amount of entered GRP78 in the cells reached the peak within 30 min. Combing established mechanisms regarding internalization of proteins and our novel observations, we hypothesized that phagocytosis and clathrin, caveolin-1, and micropinocytosis-mediated endocytosis are potentially involved in internalization of GRP78. Both Mass Spectrum and molecular and cellular approaches will be used to test our hypothesis.

## RESULTS

### Secreted GRP78 can enter into macrophages

**Figure 1:**
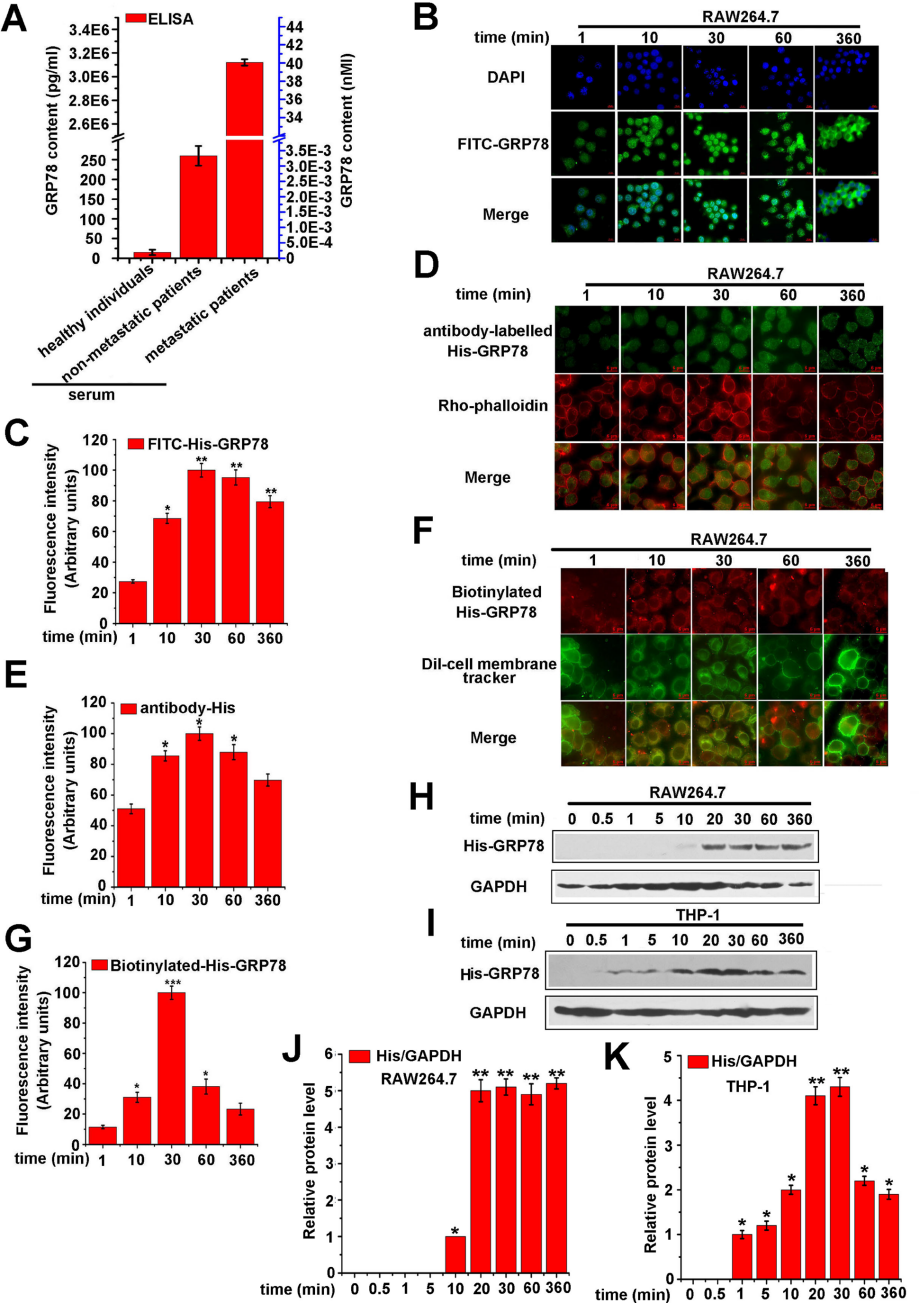
Secreted GRP78 is able to enter into macrophages (**A**) ELISA analysis of GRP78 in serums of different colorectal cancer patients. (**B**) RAW264.7 cells were treated with 40 nM FITC-GRP78 for different lengths of time at 37° C, and cellular uptake was examined using fluorescence microscopy. Green: FITC-GRP78; blue, DAPI. Red scale bars in the panels represent 10 μm. (**C**) Quantification of FITC-GRP78 fluorescence intensity was performed by Image J software (^*^p < 0.05, ^**^P < 0.01). (**D**) RAW264.7 cells were treated with 40 nM His-GRP78 at 37° C and processed for indirect immunofluorescence using anti-His and the corresponding His-conjugated secondary antibodies. Red, Rho-labeled phalloidin; Green: His-conjugated secondary antibody. Red scale bars in the panels represent 6 μm. (**E**) Average His-GRP78 fluorescence intensity of the fluorophore in each time point (^*^p < 0.05). (**F**) RAW264.7 cells were treated with 40 nM biotin-labelled GRP78 at the indicated time points at 37° C, and cellular uptake was examined using fluorescence microscopy. Green: DiI-cell membrane Tracker; Red, streptavidin-conjugated biotin-labelled-GRP78. The red scale bars in the panels represent 6 μm. (**G**) Average biotin-labelled GRP78 fluorescence intensity of the fluorophore in each time point was calculated Using Image J software (^*^p < 0.05, ^***^p < 0.001). (**H and J**) RAW264.7 cells were treated with His-GRP78, and processed for Western blot analysis. Anti-His antibody was used to determine the levels of intracellular internalized protein and mouse anti-GAPDH antibodies for protein loading control (H) and the relative protein expression was calculated by Image J (J). (**I and K**) THP-1 cells were treated as described in (H) and the relative protein expression was calculated by Image J (K).

The concentration of GRP78 in serums of colon cancer patients are rarely described before. To select a clinical concentration of GRP78, 32 serum samples from human colon cancer patients (10 healthy individuals, 11 non-metastatic patients, and 11 metastatic patients) were collected, and the concentration of GRP78 was measured. As revealed by ELISA, the concentration of GRP78 was about 40 nM in metastatic patents^,^ serum, but the data in healthy individuals and non-metastatic patients^,^ serum was much lower (Figure 1A). In addition, GRP78 is overexpressed and macrophages are aggregated in advanced tumor [21, 22]. Thus, in the following experiments, we used a purified His-tagged GRP78 protein to simulate tumor-secreted GRP78 and selected 40 nM His-GRP78 as an initial dose to determine the GRP78 internalization into macrophages.

To analyze the detailed internalization process of secreted GRP78, we added FITC-labelled His-GRP78 (40 nM) into culture medium of RAW264.7 cells. The results showed that FITC-labelled His-GRP78 translocated from extracellular to intracellular immediately (within 1 min), and presented punctate and diffused fluorescence. The amount of translocated GRP78 was constantly elevated and reached the peak at 30 min. After 30 min, the internalized GRP78 could still be detectable and maintained 360 min in cytosolic (Figure 1B and 1C). Similar to the effect that observed with FITC labelled His-GRP78, the results of biotin-labelled GRP78 and anti-His staining also presented distinct internalized phenomenon (Figure 1D, 1E, 1F and 1G). Consistently, Western blot and quantified results demonstrated that His-GRP78 could be detected largely within 20 min in lysate of RAW264.7 and THP-1 cells, and lasting 360 min (Figure 1H, 1I, 1J and 1K). Collectively, these results indicated that secreted GRP78 could enter into macrophages rapidly and kept a long period of time.

### Secreted GRP78 enters into macrophages by endocytosis

**Figure 2:**
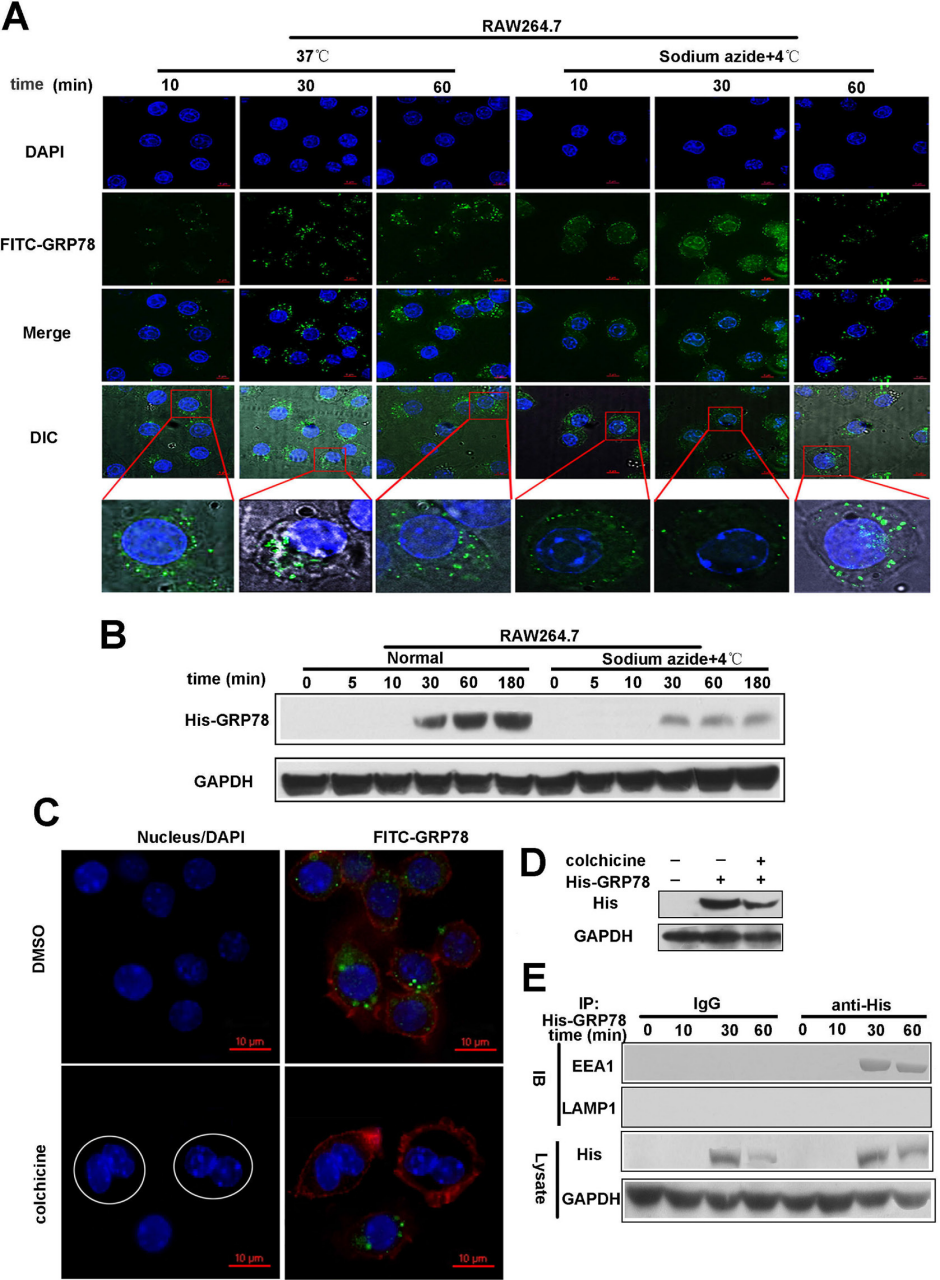
Endocytosis mediates the internalization of secreted GRP78 into macrophages (**A**) Confocal microscopic images of RAW264.7 cells incubated with 40 nM FITC-GRP78 for the indicated time intervals under energy depletion or normal cell culture. Scale bars represent 6 μm. (**B**) Western blot analysis of His-GRP78 in RAW264.7 cells. Cells treated with or without 0.1% Sodium azide were incubated with His-GRP78 for the indicated time points. (**C**) RAW264.7 cells in the M phase failed to uptake FITC-GRP78 (green). Fluorescence microscopy image for RAW264.7 cells treated with 30 μM colchicine for 16 h. Round shapes represents RAW264.7 cells that were arrested in the M phase. In the images of FITC-GRP78/colchicine, in contrast to the neighboring cells in interphase, the drug-treatment group represents cells that failed to uptake FITC-GRP78. Scale bars represent 10 μm. (**D**) The cells were pre-treated with 30 μM colchicine for 16 h and then the uptake of His-GRP78 was detected by Western blot. (**E**) Immunoprecipitation analysis of His-GRP78 in RAW264.7 cells incubated with 40 nM His-GRP78 for the indicated time intervals. His was immunoprecipitated and IgG served as a negative control, and then EEA1 and LAMP1 was examined.

Endocytosis is ATP-dependent. To elucidate whether secreted GRP78 entered into macrophages was endocytosis-depend, we used sodium azide to block ATP synthesis. As expected, cell-energy depletion virtually abolished punctate fluorescence in cytoplasm and formed small clusters of FITC-GRP78 particles attached to the cell membrane within 30 min (Figure 2A). This observation indicated that the entry of tumor secreted GRP78 into RAW264.7 cells is energy-dependent. In addition, the blockage of His-GRP78 entrance was also observed after sodium azide treatment as revealed by Western blot (Figure 2B).

Membrane traffic event is inhibited during the M (mitotic) phase in mammalian cells, therefore endocytosis is shut down in the meantime. After exposure to 100 nM colchicine for 16 h, more than 50% of RAW264.7 cells exhibited M-phase nuclear morphology and the uptake of FITC-GRP78 was shut down almost completely by immunofluorescence staining and western blot detection (Figure 2C and 2D).

More importantly, internalized His-GRP78 had obvious colocalization signal with early endosome marker EEA1 at different time points, but not late endosome marker LAMP1 (Figure 2E). These evidences supported that tumor-secreted GRP78 mainly functions by entering into cells rather than embedded in plasma membrane.

### Phagocytosis contributes to the internalization of secreted GRP78 into macrophages

**Figure 3:**
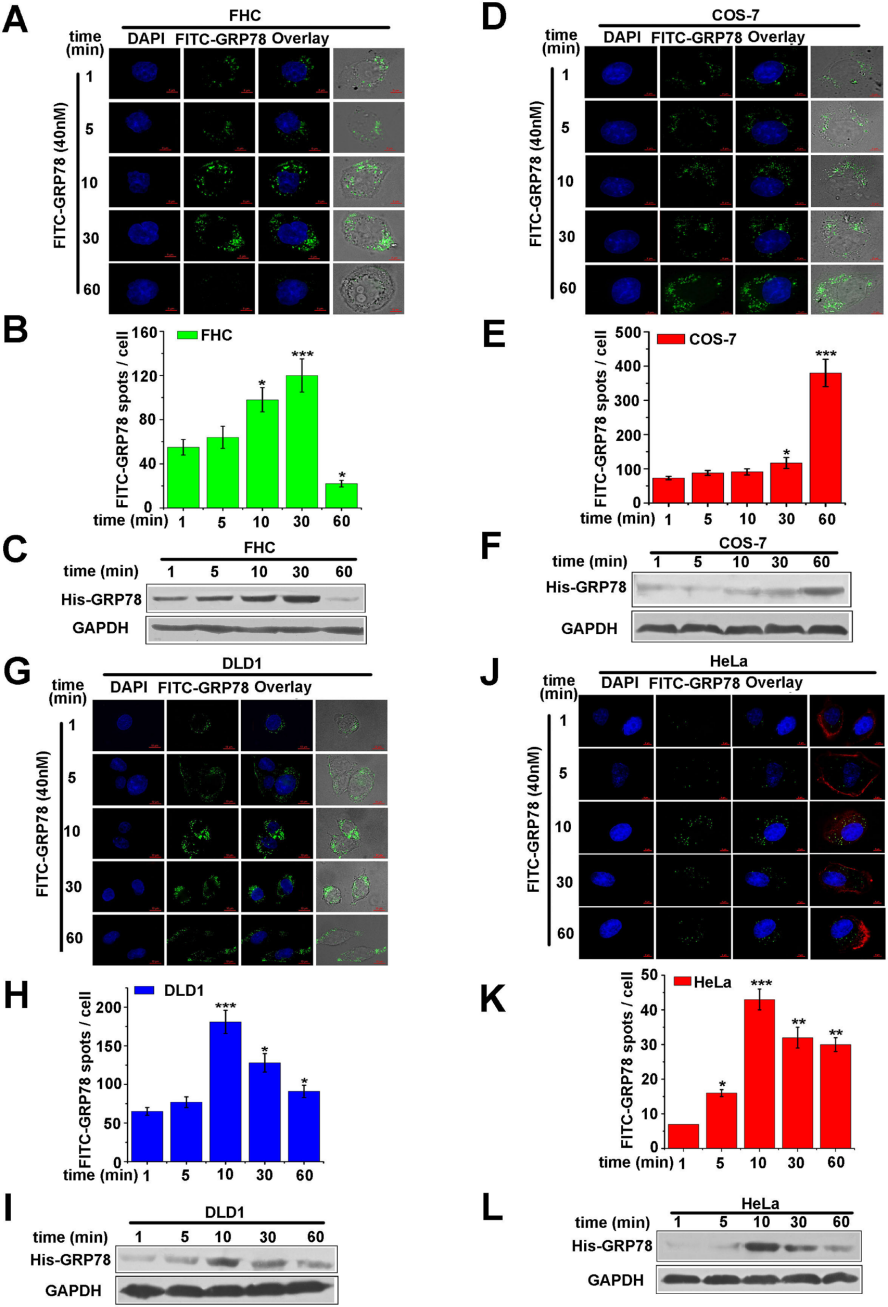
Phagocytosis is not the only way for secreted GRP78 to enter into macrophages (**A, D, G, J**) Location of GRP78 in FHC, COS-7, DLD1, HeLa cells treated with 40 nM FITC-GRP78 at different time points. Corresponding images were superimposed to determine the location of GRP78. Scale bars represent 6 μm. (**B, E, H, K**) Concentration of GRP78 protein in the above cells. As described in (A, D, G, J), experiments were repeated three times, and 250 cell per time point in each experiment were scored in the quantification analysis using Image J software. (**C, F, I, L**) Levels of internalized protein. As described in (A, D, G, J), Western blot data were superimposed to determine the levels of internalized protein. Mouse anti-GAPDH antibodies was used as a loading control.

Phagocytosis is a specialized uptake route for macrophages, which enables macrophages to take up foreign particles. Therefore, we speculate that the internalization of secreted GRP78 in macrophage was mediated by phagocytosis. To test this hypothesis, purified His-GRP78 were applied to other cell lines (including two types of cancer cells HeLa and DLD1, two normal cell lines of COS-7 and FHC) to analyze whether secreted GRP78 could enter into these cells. In detail, FITC-labeled His-GRP78 protein was used to treat cells described above. The quantitative results showed that the amount of internalized FITC-GRP78 at different time points and the time points at which FITC-GRP78 reached the maximum uptake amount were different in different cell lines (Figure 3B, 3E, 3H and 3K), but FITC-labelled His-GRP78 could enter into all of these cells (Figure 3A, 3D, 3G and 3J). Likewise, the entered His-GRP78 are also detectable in the lysates as revealed by Western blot (Figure 3C, 3F, 3I and 3L). All these results demonstrated that in addition to phagocytosis, other(s) endocytosis pathway(s) also contributes to the internalization of secreted GRP78 into macrophages.

### Internalization of secreted GRP78 is dependent of macropinocytosis

**Figure 4:**
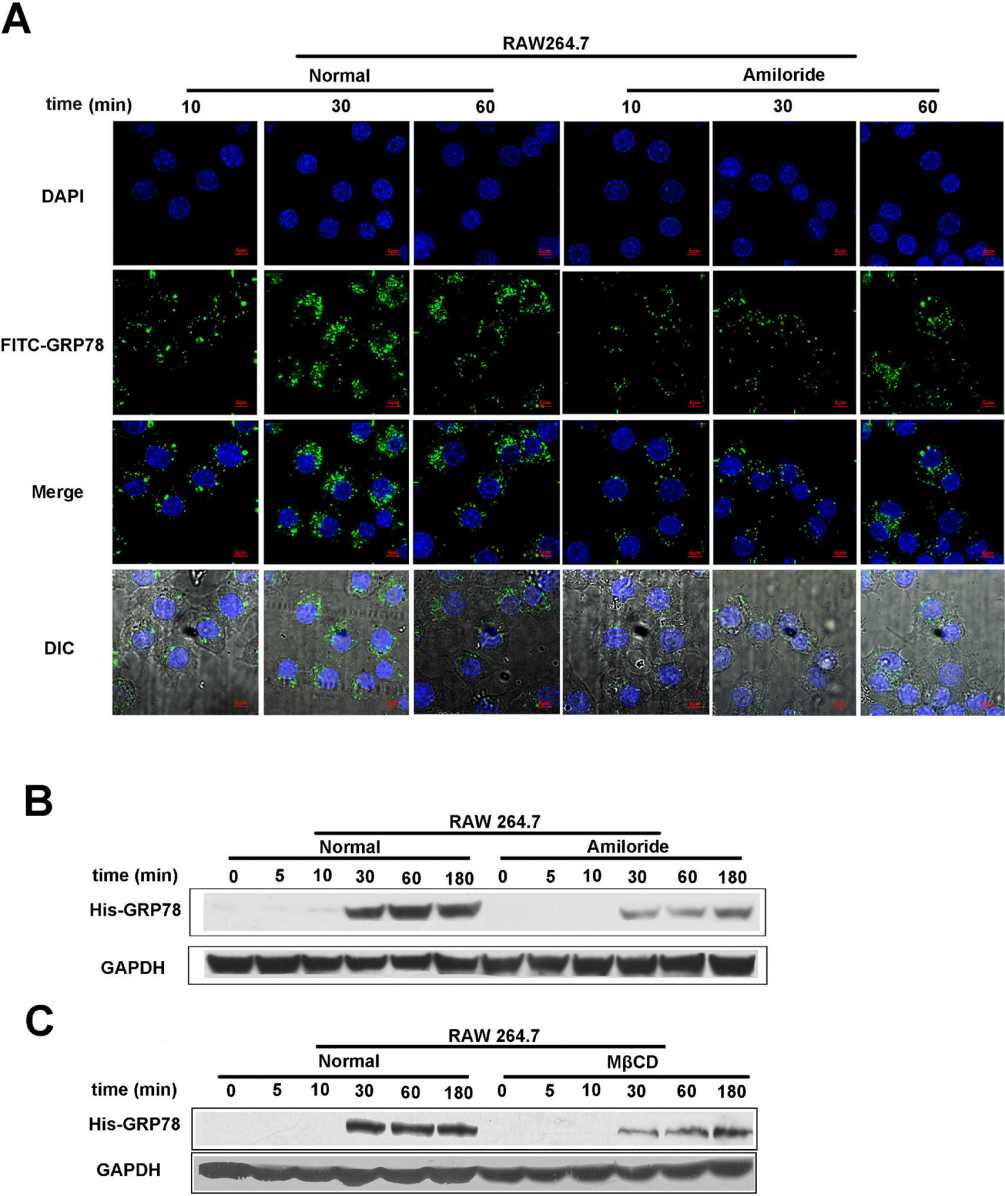
Macropinocytosis contributes to secreted GRP78 internalization (**A**) Confocal microscopic images of RAW264.7 cells incubated with 40 nM FITC-GRP78 for the indicated time intervals in the condition of untreated or pretreated with 50 μM Amiloride for 45 min. Experiment was repeated three times. Scale bars represent 6 μm. (**B**) Levels of internalized protein in cells were treated as described in (A). Western blot was used to determine the level of internalized protein and mouse anti-GAPDH antibodies served as a loading control. (**C**) Levels of internalized protein. Cells were incubated with 40 nM His-GRP78 for the indicated time intervals in the condition of untreated or pretreated with 5 mM MβCD for 60 min.

Phagocytosis is not the only approach for the internalization of secreted GRP78 into macrophages. Macropinocytosis is a constitutively activated process in macrophages [19]. Based on our observation and those of others, we posit that this non-selective endocytic mechanism potentially mediates the entry of secreted GRP78 into RAW264.7 cells. To test this hypothesis, amiloride, an inhibitor of micropinocytosis that impair Na^+^/H^+^ exchange [23], was used to examine whether secreted GRP78 entered into macrophages by this pathway. As shown in Figure 4A, FITC-GRP78 vesicles were trapped in the cytoplasm by stimulation with FITC-GRP78 for 10 min in control group, but few FITC-GRP78 vesicles were detected in cytoplasm after amiloride treatment. Likewise, a marked decrease in His-GRP78 was observed in amiloride-treated cells by Western blot (Figure 4B). Consistent with amiloride treatment, MβCD, another inhibitor of micropinocytosis also attenuated the internalization of His-GRP78 (Figure 4C). These results provided a strong evidence that amiloride/MβCD treatment efficiently inhibited uptake of His-GRP78 in RAW264.7, and the secreted GRP78 could be internalized in a micropinocytosis-dependent manner.

### Secreted GRP78 entry into macrophages is also dependent on clathrin- and caveolin-1-mediated endocytosis

**Figure 5:**
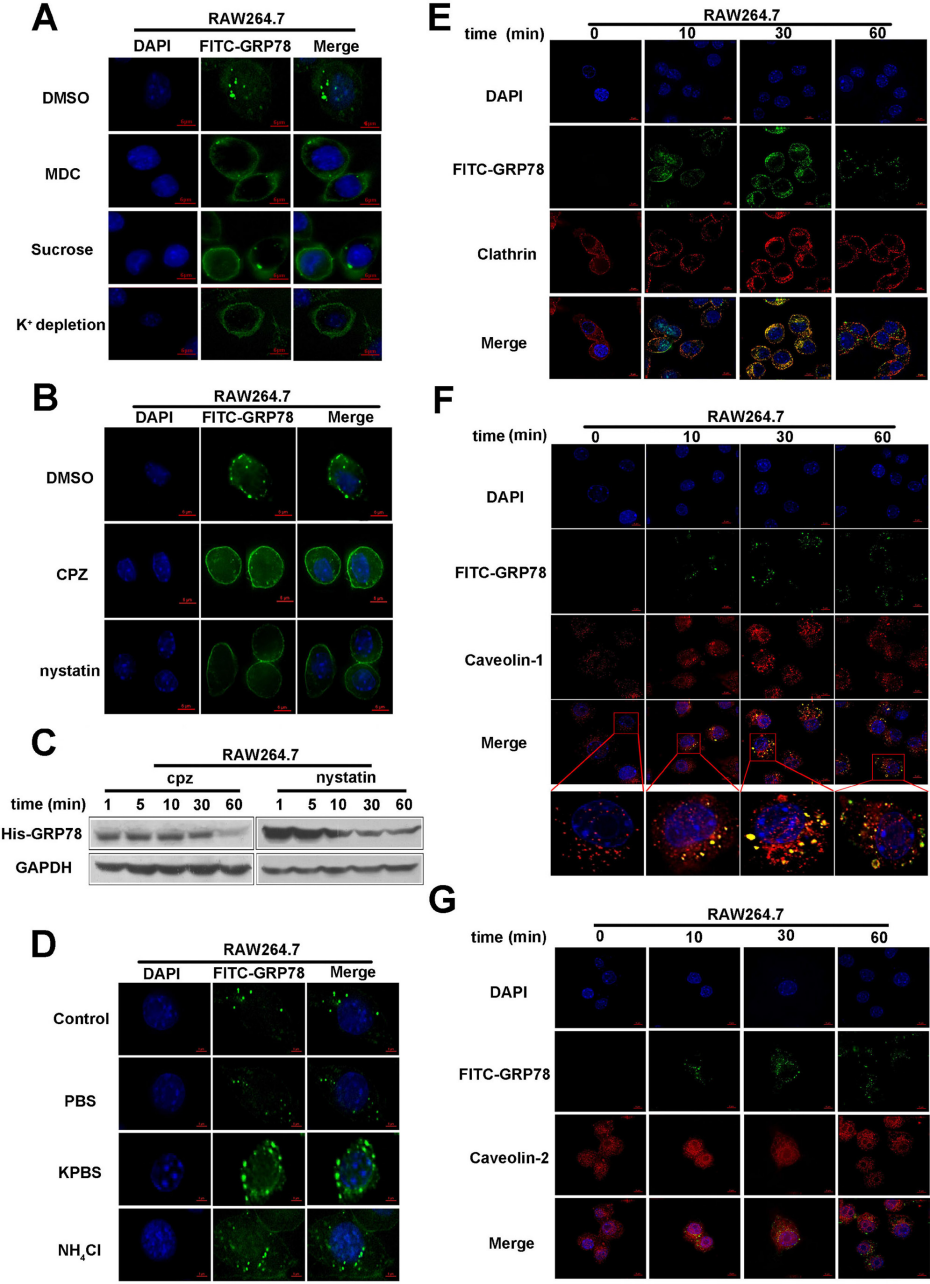
Clathrin- and caveolin-1 mediate the internalization of secreted GRP78 into macrophages (**A**) Immunofluorescence analysis of FITC-GRP78 in RAW264.7 cells. In detail, cells were pre-treated with hypertonic sucrose (0.4 M) and MDC (100 μM) or subjected to K^+^ depletion, following incubation with 40 nM FITC-GRP78. After removal of un-entered FITC-GRP78 by washing, internalized FITC-GRP78 was analyzed by immunofluorescence staining. Scale bar represents 6 μm. (**B**) Internalization analysis of FITC-GRP78 in RAW264.7 cells treated with or without 100 μM of Nystatin or 30 μM of CPZ. After incubation with FITC-GRP78, cells were washed to remove the unentered FITC-GRP78 and immunofluorescence staining was used to determine FITC-GRP78 in RAW264.7 cells. Scale bar represents 6 μm. (**C**) Protein level analysis in RAW264.7 cells that were treated as described in (B). Western blot was used to determine His in whole-cell lysates. (**D**) Location of GRP78 in live RAW264.7 cells were incubated with 40 nM FITC-GRP78 in PBS, KPBS, NH_4_Cl, or alone for 5 min. The live cells were observed under a confocal microscope. Scale bar represents 6 μm. (**E, F and G**) Location of GRP78 in RAW264.7 cells treated with 40 nM FITC-GRP78 for different time intervals and processed for indirect immunofluorescence using anti-Clathrin (E), anti- Caveolin-1 (F) or anti- Caveolin-2 (G) and the corresponding HRP-conjugated secondary antibodies. Then cells were fixed and analyzed using Delta Vision. Scale bar represents 6 μm.

Clathrin-mediated endocytosis (CME) is an important endocytic pathway for mammalian cells [17]. To examine whether the entrance of secreted GRP78 into RAW264.7 cells was also CME dependent, three kinds of inhibitors, hypertonic sucrose, MDC and K^+^ depletion, were used to block the formation of clathrin-coated vesicle [24–26]. The results showed that these inhibitors treatment led to a noticeable shift from a puncta distribution of FITC-GRP78 in cytoplasmic to a diffused distribution on plasma membrane (Figure 5A). Additionally, chlorpromazine (CPZ), a cationic amphiphilic molecule, could also disrupt the assembly of clathrin lattices at the cell surface [27], was used to confirm the Clathrin-mediated endocytosis. The results of immunofluorescent staining and Western blot showed that CPZ impaired GRP78 internalization (Figure 5B and 5C). All of these results indicated that Clathrin-mediated endocytosis contributed to the internalization of secreted GRP78.

Next, to specifically test whether secreted GRP78 is dependent on a caveolin/lipid rafts-mediated pathway, we used nystatin to interfere cholesterol synthesis to disrupt lipid rafts. As shown in Figure 5B, the nystatin treatment resulted in appearance of diffused fluorescence in plasma membrane, but not punctate fluorescence. Similar as the results from fluorescence staining, Western blot also revealed that the internalized GRP78 was reduced after nystatin treatment (Figure 5C). This visible reduction in His-GRP78 also suggested that secreted GRP78 could enter into RAW264.7 cells via caveolin/lipid rafts-mediated pathway.

NH_4_Cl and KPBS could suppress the release of macromolecules from early endosome to the cytoplasm by neutralizing the acid of endosomes [28, 29]. Therefore, these two inhibitors are used to block the clathrin- and caveolin-mediated endocytosis, the results showed that the size of the detectable FITC-GRP78 vesicles is larger in experimental group than that in control group (Figure 5D). Thus, we confirmed that secreted GRP78 internalization dependence of Clathrin/ caveolin-mediated endocytosis.

**Figure 6:**
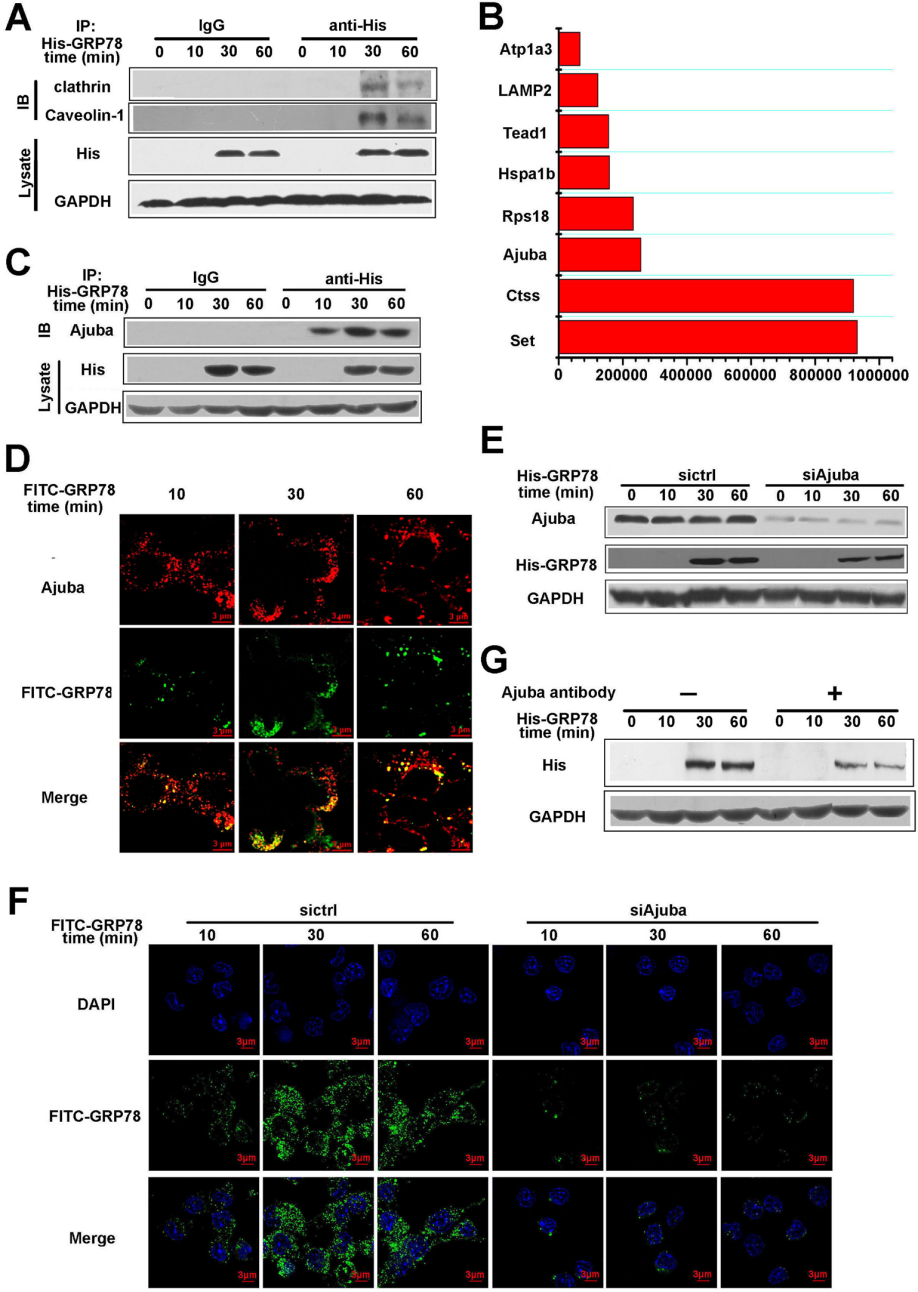
Ajuba served as a receptor to mediate the entry of secreted GRP78 into macrophages (**A**) RAW264.7 cells were treated with 40 nM His-GRP78 for different time courses. His was immunoprecipitated and IgG served as a negative control, Clathrin and Caveolin-1 was then examined. (**B**) Quantification of the top eight proteins in 191 proteins detected by MS. Western blot was used to determine expression of Ajuba in RAW264.7 cells treated with 40 nM His-GRP78. (**C**) RAW264.7 cells were treated with 40 nM His-GRP78 for different time courses. His was immunoprecipitated and IgG served as a negative control, Ajuba was then examined. (**D**) RAW264.7 cells were treated with 40 nM FITC-GRP78 for different time course and processed for indirect immunofluorescence using anti-Ajuba and the corresponding CY5-conjugated secondary antibodies. Then cells were fixed and analyzed using Delta Vision. Scale bar represents 3 μm. (**E**) Levels of Ajuba in RAW264.7 cells transfected with control siRNA or siRNA targeting Ajuba. Cells were exposed to 40 nM His-GRP78 for different time points. 48 h later, the levels of Ajuba protein in cells were examined by Western blot. (**F**) GRP78 levels in RAW264.7 cells after Ajuba knockdown. Cells were transfected with Ajuba siRNA or control scramble. 48 h later, cells were exposed to 40 nM FITC-GRP78 for different lengths of time. Cells were fixed and examined by fluorescence microscopy. Scale bar represents 3 μm. (**G**) His-GRP78 levels in RAW264.7 cells after Ajuba antibody blockage. Cells were treated with Ajuba antibody or IgG. 24 h later, cells were exposed to 40 nM His-GRP78 for different lengths of time, the levels of His protein in cells were examined by Western blot.

We then detected the co-localization of FITC-positive vesicles with Clathrin, Caveolin-positive vesicles, and found that FITC-positive vesicles co-localized with clathrin and caveolin-1 - positive vesicles, but not caveolin-2 - positive vesicles (Figure 5E–5G, Supplementary Videos 1–8). Consistent with this, the interaction between His-GRP78 and Clathrin/Caveolin-1 was also detected by Co-IP (Figure 6A). Taken together, these results suggested that the internalization of secreted GRP78 relied on different endocytic mechanisms, and the escape of GRP78 from vesicles to the cytosol was acidity endosomal dependent.

### Ajuba is a receptor that mediates secreted GRP78 entry into macrophages

All endocytic pathways of GRP78 entrance described above were cell-surface receptor-dependent except macropinocytosis. To identify the potential signal receptor(s), RAW264.7 cells were treated as described in the Section ‘The seeking of receptor that mediate secreted GRP78 entry into macrophages’ in Methods, and then the elution was subjected to mass spectrometry (MS). MS detected 191 proteins that are relatively abundant, we chose the top eight abundant proteins as the candidate receptors that mediates the entry of secreted GRP78 into macrophages (Figure 6B). The correlated properties of these eight proteins were listed in Table 1. Combining with the previous study, we identified Ajuba as the only protein that locates in plasma membrane. Thus, we speculated that Ajuba might be the receptor that mediates GRP78 internalization. Indeed, Co-IP and immunofluorescence staining revealed that His-GRP78 and Ajuba not only co-localized on plasma membrane, but also interacted with each other (Figure 6C and 6D). To further validate the role of Ajuba in facilitating GRP78 internalization, we knocked down Ajuba using its siRNA. The results showed that ablation of Ajuba impaired the entry of His-GRP78 into cells (Figure 6E and 6F). The similar results were obtained through using Ajuba antibody to block the role of Ajuba in the membrane (Figure 6G). The above data indicated that Ajuba was the receptor that mediates secreted GRP78 entry into macrophages.

**Table 1:** Mass spectrometry analysis of GRP78 binding proteins

Gene name	Protein ID	Pep count	Unique pep count	Cover percent	MW	PI
*Set*	Q9EQU5	2	2	10.70%	24923.34	5.43
*Ctss*	Q3UD32	1	1	2.06%	38456.16	6.32
*Ajuba*	Q91XC0	3	3	15.65%	57919.98	7.14
*Rps18*	P62270	1	1	7.24%	17746.46	11.1
*Hspa1b*	P17879	3	3	5.77%	70078.31	5.53
*Tead1*	Q3USK5	1	1	2.92%	46370.38	8.78
*LAMP2*	Q8C5K0	1	1	1.93%	45752.57	6.7
*Atp1a3*	Q8VCE0	1	1	2.01%	49572.88	5.17

The correlated properties of the top eight abundant proteins from MS detected 191 proteins.

### Internalized secreted GRP78 locates in ER and mitochondria of macrophages

**Figure 7:**
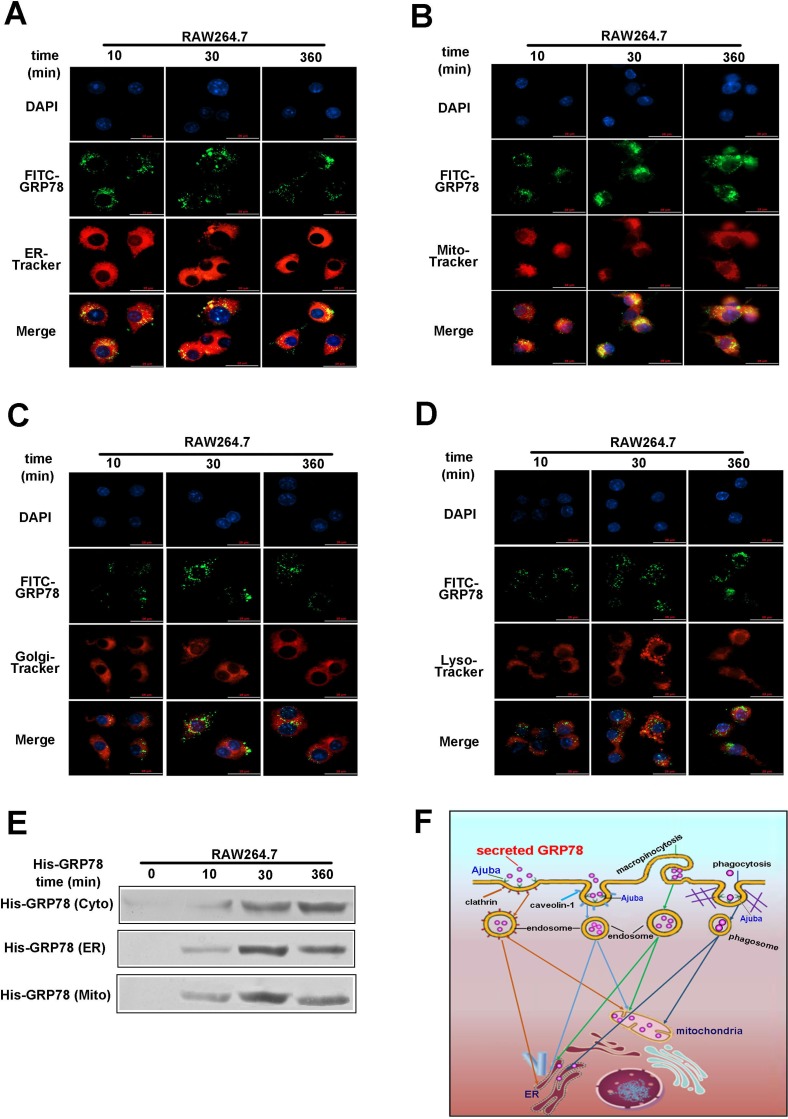
Internalized secreted GRP78 located in ER and mitochondria in macrophages (**A–D**) Colocalization analysis of GRP78 in RAW264.7 cells treated with His-GRP78 for different lengths of time. Mitochondria, ER, Golgi and lysosome were labelled with Mito-Tracker Red dye, ER-Tracker Red dye, Golgi-Tracker Red dye and Lyso-Tracker Red dye, respectively. Colocalization analysis was performed using confocal microscopy. Scale bar represents 25 μm. (**E**) Protein levels of GRP78 in fractions of ER and mitochondria fractions isolated from RAW264.7 cells with or without His-GRP78 treatment for different lengths of time. After cell lysis, ER and mitochondria fractions were subjected to Western blot analysis to probe for His. (**F**) A schematic representation of the proposed model. Tumor secreted GRP78 is internalized into macrophages predominately through phagocytosis, clathrin-, caveolin-1 and micropinocytosis-mediated endocytosis and then located into the mitochondrion and ER, lasting 6 h at least.

To uncover the subcellular localization of secreted GRP78 in macrophages, we studied the co-localization of FITC-GRP78 with ER, mitochondria, lysosomes and Golgi. Inspection under a Delta Vision microscope demonstrated that GRP78 aggregates were located in the ER and mitochondria rather than in lysosomes or Golgi (Figure 7A, 7B, 7C and 7D). To further validate the accumulation of GRP78 in the ER and mitochondria, ER and mitochondria fractions were isolated from His-GRP78 -treated cells. As expected, His-GRP78 were detected in both ER and mitochondria fractions (Figure 7E). All these results uncovered that the internalized GRP78 could translocate into the mitochondrion and endoplasmic reticulum for more than 6 h.

## DISCUSSION

Tumor-associated macrophages (TAMs) are well-described promoters of cancer progression in TME [30]. Previous study has shown that secreted GRP78 could induce polarization of mouse macrophage to M2 type. However, the mechanism of this process in macrophages is poorly described. In this study, we established an *in vitro* model to elucidate the mechanism of the internalization of secreted GRP78 by purifying a recombinant endo-free His-GRP78. Combining with immunofluorescence staining and Western blot approaches, we demonstrated that cancer cell-derived GRP78 could enter into macrophages rather than stay on the membrane to trigger cellular signaling. Furthermore, cytoplasmic GRP78 could promote tumor cell proliferation, immune escape and angiogenesis [4]. Thus, we speculate that internalization of GRP78 into macrophages may play a parallel role in improving the ability of macrophages to promote tumor progression. That is, tumor-secreted GRP78 acts as a key factor to regulate tumor microenvironment by working on macrophages.

Our results demonstrated that tumor secreted GRP78 was internalized into macrophages through different endocytic pathways. Intriguingly, macrophages internalize secreted GRP78 not only via specialized phagocytosis, but also clathrin-dependent endocytosis, caveolin-dependent endocytosis and micropinocytosis. For macrophages, phagocytosis is the unique endocytosis, so phagocytosis can also be used to uptake secreted GRP78. It has been confirmed that particles (lesser than 80 nm) were preferentially internalized by clathrin-mediated endocytosis, while particles whose sizes are between 80 nm and 200 nm, a shift toward caveolin-mediated endocytosis was needed [17, 18]. According to our data, the sizes of FITC-labelled His-GRP78 vesicles were different. Therefore, various pinocytosis pathways may function simultaneously. Moreover, we deduct that secreted GRP78 may gather and form different sizes of vesicles to exhibit a clustering distribution on macrophages cell-surface, and further internalizing into macrophages via clathrin or caveolin-mediated endocytosis. Regarding the micropinocytosis, it is primarily responsible for nonspecific uptake of fluid and smaller particles that are attached to the plasma membrane. Therefore, micropinocytosis can also be used to uptake secreted GRP78.

Except macropinocytosis, all the current reports described that endocytic pathways of GRP78 entrance were cell-surface receptor-dependent. Hence, it is critical to identify some receptors that mediate the entry of secreted GRP78 into macrophages. Combining a novel proteomic approach and other biochemical strategies, we validated Ajuba as a binding partner of secreted GRP78. The Ajuba are characterized by the presence of three highly related tandem LIM domains at their carboxyl terminus (the LIM region), and a variable proline-rich amino-terminal prelim region, and mainly locate in cytoplasm and nucleus, sometimes escape to cell-surface. Because of the virtue of its structure and subcellular localization, Ajuba functions as (1) adapters and scaffolds in the transduction of external signals such as growth factor, and (2) transcriptional co-repressors to control gene expression [31–33]. As mentioned above, Ajuba has diverse subcellular distribution and corresponding functions, but few literatures described its function as a receptor. Here, we validated that Ajuba mediates the internalization of GRP78 e into macrophages as a receptor. Ajuba’s function as a receptor was the first report in this study.

Internalized GRP78 could locate in ER and mitochondria of macrophages for more than 6 h (Figure 7A and 7B). However, the function of tumor secreted GRP78 located in ER and mitochondria need to further clarified. According to previous data, mitochondria are the primary places where metabolism occurs. And our group has reported that PKM2 depletion induces the compensation of glutaminolysis through β-catenin/c-Myc pathway in tumor cells [34]. Hence, we deduct that secreted GRP78 locates in mitochondria of macrophages may function through regulating glutaminolysis metabolism via suppressing PKM2 expression. Moreover, GRP78 was also implicated in regulation of mitochondria energy homeostasis [1, 5], it was consistent with our recent study that the glycolysis was reduced after GRP78 treatment. We thereby hypothesized that secreted GRP78 could potentially regulate mitochondrial function, such as maintaining energy and mitochondria homeostasis. ER has the KDEL retrieval system and GRP78 has KDEL signal peptides [35], further providing the evidence that secreted GRP78 could locate in ER of macrophages. GRP78 in ER is involved in many types of cellular processes, including translocating newly synthesized polypeptides, facilitating the folding and assembly of newly synthesized proteins by recognizing unfolded polypeptides and by preventing intra- or intermolecular aggregation, maintaining them in a state competent for subsequent folding and oligomerization, targeting misfolded proteins for proteasome degradation, regulating calcium homeostasis and serving as a sensor for ER stress [36]. Thus, we deduct that secreted GRP78 located in ER may play the similar roles in regulator these cellular processes.

Tumor-secreted GRP78 could enter into RAW264.7 and THP-1 cells without locating on the plasma membrane to transfer signals into cells. By using pharmacological inhibitors to interrupt the function of different endocytic axis, we confirmed that both phagocytosis and clathrin, caveolin-1 and micropinocytosis-mediated endocytosis pathways are involved in the internalization of secreted GRP78 into cells (Figure 7F). Furthermore, we identified Ajuba as a receptor that was responsible for secreted GRP78 internalization into RAW264.7 cells (Figure 7F). Finally, our study established that internalized GRP78 could escape from endosomes, and translocate into the mitochondrion and endoplasmic reticulum for more than 6 h (Figure 7F). This study not only reveals an pro-survival strategy of tumor cells which secrete proteins to swiftly educate stromal cells such as macrophages to form an adaptive microenvironment, but also provides theoretical foundation for improving stroma-targeted therapies in cancer treatment.

## MATERIALS AND METHODS

### Reagents and antibodies

RPMI-1640 medium, Dulbecco^,^s modified Eagle^,^s (DMEM) medium, DMEM / F-12 1:1 medium and fetal bovine serum (FBS) were from GIBCO (Grand Island, NY). Colchicine, sodium azide, Amiloride, monodansylcadaverin (MDC), sucrose, Hepes, ammonium chloride (NH_4_Cl), chlorpromazine(CPZ) and Streptavidin-Agarose were obtained from Sigma (St. Louis, MO). Nystatin was from Sangon Biotech (Shanghai, China). His antibody was from Abcam (Cambridge, UK). Antibodies for clathrin and Caveolin-1 were obtained from Proteintech (Chicago, USA). GAPDH was from Bioworld Technology (Minneapolis, MN). Caveolin-2 antibody was obtained from Bioss (Peking, China). Antibodies against LAMP1 and EEA1 were purchased from Cell Signaling Technology (Danvers, USA). FITC- and TRITC- secondary antibodies as well as ER-Tracker, Mito-Tracker, Lyso-Tracker and DTSSP were obtained from Thermo (Carlsbad, CA). Golgi-Tracker Red and DiI were purchased from Beyotime Institute of Biotechnology (Shanghai, China). FITC- and biotin- labelling kits were obtained from Elabscience Biotechnology Co.,Ltd (Wuhan, China). PE-streptavidin was from Biolegend (California, USA). Rhodamine was obtained from Cytoskeleton, lnc. (Colorado, USA).

### Cell culture

Human colon carcinoma DLD1, human cervical cancer HeLa, African Green Monkey kidney COS-7 and human normal colon epithelial FHC cell lines were obtained from the American Type Culture Collection and cultured in RPMI-1640 medium supplemented with 10% FBS and 1% penicillin-streptomycin at 37° C in a humidified tissue culture incubator containing 5% CO_2_. Human normal colonic mucosa FHC was cultured in DMEM / F12 1:1 medium supplemented with 10% FBS, and the murine macrophage RAW264.7 was cultured in DMEM medium supplemented with 10% FBS.

### His-GRP78 labelling

His-tagged GRP78 was produced in accordance with our laboratory protocol [37]. Purified His-GRP78 was labelled with fluorescein FITC/biotin according to the manufacturer’s instructions (Elabscience).

### Tumor secreted GRP78 internalization assay

RAW264.7 cells were cultured on glass coverslips. Fluorescently conjugated His antibody/FITC-labeling His-GRP78/ biotin-labeling His-GRP78 was incubated with cells at 37° C for various lengths of time to allow molecules internalization. To remove unbound antibody, cells were washed three times in phosphate-buffered saline (PBS). Then the cells were fixed, and processed for immunofluorescence as previously described [37].

For experiments to monitor internalization of cell-energy depletion, 45 min pre-incubation of cells in 0.25% NaN_3_ in HEPES buffer on ice was required, then followed by incubation with the same incubation buffer containing labelled FITC-GRP78 in DMEM media containing 20 mM HEPES.

To quantify the difference in uptake of labelled GRP78 between interphase and M phase in RAW264.7 cells, the cells arrested in M phase with colchicine treatment (30 μM for 16 h) were carried out.

Two buffers of KPBS and PBS were used for membrane potential elimination. PBS is composed of 136.9 mM NaCl, 2.7 mM KCl, 30 mM Na_2_HPO_4_, 1.76 mM KH_2_PO_4_ with 0.1% glucose at pH 7.4. KPBS had the same composition as PBS, with the exception that KCl was used to replace NaCl. To test the effect of membrane potential, RAW264.7 cells maintained in 12-well plates at 70% confluence were washed with PBS or KPBS and then incubated with 40 nM FITC-GRP78 for endocytosis in PBS or KPBS buffer, respectively. Endocytosis of GRP78 was determined by Delta Vision.

### Manipulation of endocytic pathways by chemical inhibitors

RAW264.7 cells maintained on polylysine-coated overslip were pre-treated with 0.45 M sucrose, 100 μM MDC, 50 mM NH_4_Cl, 50 μM Amiloride for 1 h and 50 μM nystatin, 30 μM CPZ for another 15 min at 37° C. The K^+^ depletion was carried out according to the procedure described by Altankov and Grinnell (1993). Briefly, cells were rinsed once with potassium-free buffer (140 mM NaCl, 20 mM HEPES, 1 mM CaCl_2_, 1 mM MgCl_2_ and 1 mg/mL D-glucose, at pH 7.4). Cells were incubated in hypotonic medium (50% potassium-free buffer/50% H_2_O) for 30 min at 37° C, and then incubated in potassium-free buffer for another 10 min at 37° C. Cells that were treated with different inhibitors were then incubated with FITC-GRP78 at 37° C for 10 min to facilitate endocytosis. After 10 min of incubation, cells were placed on ice to terminate the reaction. Inhibition of endocytosis was confirmed by confocal microscopy and quantified by Image J.

### The seeking of receptor that mediate secreted GRP78 entry into macrophages

RAW264.7 cells, with two plates per group, grown in 100 mm plates at 90% confluence were treated with 40 nM biotinylated-His-GRP78 in two treatment groups for 30 min. After two times washing of PBS, crosslinker-DTSSP solution was added into cells to a final concentration of 2 mM. After incubation of the reaction mixture at room temperature for 30 min, stop solution (1 M Tris, Ph7.5) was added into the reaction mixture to a final concentration of 20 mM. After cross-linking reaction for 15 min, cells were lysed and extraction were incubated with streptavidin-agarose beads for 2 hours at 4° C by gently shaking. Unbound protein was removed by PBS washing and biotinylated proteins were eluted using 0.1 M glycine HCl (pH 2.5). Eluted protein samples were immediately neutralized using 1M Tris (pH8.0) and subjected to mass spectrometry.

### RNA interference

Small interfering RNA (siRNA) of mouse *Ajuba* and siGenome non-targeting siRNA (control) were used for *Ajuba* knockdown. Ajuba siRNAs: sense 5′-GCGUCAAUGGCUCUGUCUATT-3′, and anti-sense 5′-UAGACAGAGCCAUUGACGCTT-3′. A negative control siRNA: sense 5′-UUCUCCGAACGUGUCACGUTT-3′, and antisense 5′-ACGUGACACGUUCGGAGAATT-3′) were obtained from GenePharma. Briefly, cells were grown in 12-well or 60-mm plates and transiently transfected at 60–80% confluence with siRNA at a final concentration of 5 nM using siRNA-Mate™ transfection reagent (GenePharma) according to the manufacturer^’^s instructions. After 48 h transfection, the cells were incubated with fresh medium alone or with indicated concentration of His-GRP78/FITC-GRP78 for different lengths of time. Then cells were used for immunofluorescence and Western blot analysis.

### ER and mitochondrion isolation

An ER isolation kit (Sigma) was used to isolate ER according to the manufacturer’s instructions. Briefly, 5 × 10^8^ cells were treated with GRP78 at different time intervals, and then harvested, re-suspended in hypotonic buffer at a volume three times that of the packed pellet. Cells were homogenized using a Dounce homogenizer. Cell extracts were then subjected to a discontinuous iodixanol gradient by centrifugation at 12,000 × g for 15 min and 100,000 × g for 16 h to obtain mitochondrion and ER fractions, respectively. Mitochondrion and ER fractions were collected, solubilized in Western buffer and analyzed by Western blotting.

### Western blot and immunoprecipitation

Cells were lysed in Western and IP buffer (Beyotime, China) containing a protease inhibitor cocktail (Thermo scientific). 800 μg supernatant from whole-cell lysates (a final volume of 1 ml) were pre-incubation with 1.5 μg control IgG corresponding to the host species of the primary antibody and immunoprecipitation antibodies overnight followed by incubation with 50 μl of Protein A/G PLUS-Agarose (Santa Cruz) at 4° C for 2 h. Beads were isolated by centrifugation at 4° C for 5 min at 2,500 rpm. After three times washing with cell lysis buffer, beads were boiled in 2 × SDS loading buffer for 5 min, and supernatants were loaded on SDS-PAGE and subjected to Western blot analysis, as previously described.

### Immunofluorescence

Cells were seeded onto 6-well glass slides. After treatment, cells were fixed in 4% paraformaldehyde in PBS for 30 min and permeabilized with 0.3% Triton X-100 in PBS for 10 min. Slides were then blocked in 2% goat serum for 1 h and incubated with primary antibodies at 4° C overnight. After incubation, slides were then washed and incubated with the corresponding secondary antibodies. After three times of PBS washing, slides were mounted in gelvatol for confocal immunofluorescence analysis.

### Statistical analysis

Data represent the mean ± SEM. Statistical significances among groups were tested by a one-way analysis of variance (ANOVA), and comparisons between two groups were evaluated using Student *T*-test. *p* < 0.05 was considered statistically significant.

## SUPPLEMENTARY MATERIALS VIDEOS



















## Abbreviations

GRP78: Glucose-regulated protein 78
TAMs: tumor-associated macrophages
TME: tumor microenvironment
SDS-PAGE: Sodium dodecyl sulfate polyacrylamide gel electrophoresis
PVDF: polyvinylidene fluoride
MDC: monodansylcadaverin
NH_4_Cl: ammonium chloride
CPZ: chlorpromazine
