# Supplementary material for: Ajuba receptor mediates the internalization of tumor-secreted GRP78 into macrophages through different endocytosis pathways
Source: Oncotarget. 2018 Jan 9;9(21):15464–79. doi: 10.18632/oncotarget.24090 (PMC5884641; doi:10.18632/oncotarget.24090)
Supplement: Supplementary file 1 [file oncotarget-09-15464-s001.pdf]

Ajuba receptor mediates the internalization of tumor-secreted GRP78 into macrophages through different endocytosis pathways

SUPPLEMENTARY MATERIALS

Supplementary Videos 1–8: FITC-GRP78 and Clathrin/Caveloin-1-positive structures were analyzed by Delta Vision. As described in Figure 5, RAW264.7 cells treated with His-GRP78 for different time points were mounted on coverslips and subjected to Delta Vision. Final visualization and video production were performed in SoftWoRx using selected cropped regions of the data sets. Note that vesicles positive for green and red appears yellow. See Supplementary_Videos_1–8
